# Isotropic ordering of ions in ionic liquids on the sub-nanometer scale†
†Electronic supplementary information (ESI) available. See DOI: 10.1039/c7sc05184k


**DOI:** 10.1039/c7sc05184k

**Published:** 2017-12-22

**Authors:** Hailong Chen, Xin Chen, Jingwen Deng, Junrong Zheng

**Affiliations:** a College of Chemistry and Molecular Engineering , Beijing National Laboratory for Molecular Sciences , Peking University , Beijing 100871 , China . Email: junrong@pku.edu.cn ; Email: zhengjunrong@gmail.com; b Beijing National Laboratory for Condensed Matter Physics , CAS Key Laboratory of Soft Matter Physics , Institute of Physics , Chinese Academy of Sciences , Beijing 100190 , China

## Abstract

This article investigates structures of ionic liquids. Using a high-powered multiple-mode two dimensional infrared spectroscopic technique, we measure the anisotropy of interionic vibrational coupling in ionic liquids. Such anisotropy reports the relative orientation between cations and anions. Surprisingly, opposite to the well-propagated idea of ion pairing, a random orientation between the nearest non-spherically symmetric cation and anion is observed in ionic liquids. On the one hand, numerous previous experiments and theoretical calculations have shown that ionic liquids are highly ordered at the mesoscale, forming a bicontinuous nanostructure of ionic domains and hydrophobic domains. On the other hand, our results clearly prove that the ion ordering within the ionic domains is essentially random. Such an ordering at the larger scale (nanometers) and a lack of ordering at the smaller scale (sub-nanometer) is very rare for most liquids, or may even be unique to ionic liquids. Herein, we propose that ionic liquids may be regarded as 3D interconnecting nanocomposites of molten-salt-like domains and molecular-liquid-like domains. Such unique structuring could explain the fact that ionic liquids, like composite materials, often possess favorable properties of both “ionic” and “molecular” components.

## Abstract 2

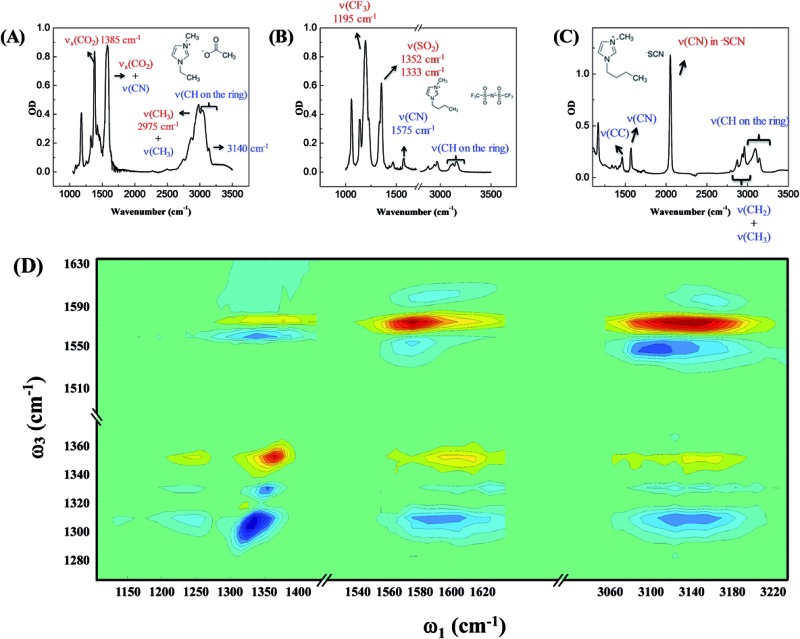
This article investigates structures of ionic liquids.

## Introduction

Among the three most common states of matter, solid, liquid, and gas, liquid is no doubt the least understood, and has been described as the “Cinderella of modern physics”.^1^ The major challenge in the study of liquids stem from it being the “intermediate” state. In liquids, molecules are both “bound” (solid-like) and “free” (gas-like). Over a small region, they can be bound by intermolecular forces and form ordered structures. Over a large range, thermal motions dominate, and liquid molecules are almost randomly distributed and thus “free”. Liquids, broadly defined, include liquid forms of molecular molecules, molten salts of ionic molecules, and ionic liquids (ILs) of a large group of compounds that are both “molecular” and “ionic”. Ionic liquids are often viewed as the “intermediate” between molecular liquids and molten salts.^2^ They enjoy many special properties available to either molecular liquids or molten salts but not both.^2^ For example, on the one hand, ionic liquids are of extremely low vapor pressure, similar to molten salts; on the other hand, ILs are good and versatile solvents, similar to molecular solvents. The chemical structures of ILs can be fine-tuned to accommodate various types of compounds, and it has been estimated there are 10^18^ possible ILs, not including any mixtures.^3^,^4^ These unique combinations of properties of molecular liquids and molten salts make ILs very attractive solvents, reaction media, or technical fluids in numerous applications, for example in batteries, solar cells, and organic synthesis.^2^–^5^

However, these superior properties of ILs cannot be obtained by blending molten salts and molecular liquids together, suggesting structures of ILs are beyond the simple mixture of two liquids. In molten salts, the inter-particle forces are mostly columbic in nature. columbic interactions are non-directional, and ions in molten salts are believed to be isotopically distributed.^2^ Whereas in molecular liquids, the inter-particle forces are mostly van der Waals (dipolar or multipolar interactions) or hydrogen bonding in nature.^2^ These forces are typically highly directional, with the notable exception of London dispersion forces for nonpolar molecules, which are important only in relatively short distances. Therefore, molecules in such liquids are almost randomly distributed over a large region, but form reasonably well-ordered structures in small distances, such as the hydrogen bond network in water. In ILs, both non-directional, relatively long-ranged columbic interactions and directional, short-ranged van der Waals interactions are important. The competition/cooperation of these interactions results in sophisticated IL structures that are responsible for their unique properties and of great interest to both theory and practice, but still subject to intense debate.^2^,^3^,^6^–^11^

Many supramolecular structures have been proposed to describe ILs, and among them the most noteworthy structures are ion pairing and ion clustering.^12^–^15^ Historically, the concept of “ion pairing” was first introduced to describe aqueous electrolyte solutions.^16^ In its classic definition, an ion pair forms if the closest neighboring cation and anion associate so strongly that the pair effectively acts like a neutral molecule with a large dipole moment. There are a handful of arguments that support the idea of “ion pairing” in ILs. In aqueous electrolyte solution, the degree of ion pairing typically increases with salt concentration.^16^–^18^ As ion pairing becomes more common and tighter in salt solutions with increasing concentration, one might expect close-contacted ions pairs in ILs if ILs were assumed to behave like highly concentrated salt solutions. Such an extrapolation, however, might be questionable,^12^ because ILs are intrinsically heterogeneous. For some ILs measured by mass spectroscopy, their ions clearly pair up in the gas phase.^19^,^20^ Another indirect supporting argument for ion pairing in ILs comes from the “unexpectedly low” bulk ionic conductivities.^21^ Experimental values of bulk ionic conductivity of ILs are often much smaller than the corresponding calculated values if one assumes ions are “free” and have unit charges.^22^,^23^ Unsurprisingly, it has long been proposed that a good portion of ions in ILs form ion pairs and do not contribute to the overall ionic conductivity.^12^,^22^ Nevertheless, very few experiments can directly demonstrate the existence of ion pairs in ILs. Some experimental studies did claim strong ion paring in ILs.^2^,^24^ The interpretations of these experimental observations, however, often rely on certain assumptions that are questionable in ILs. For example, in a 2013 PNAS publication,^6^ IL structures were measured using a scanning probe microscope on a surface. By fitting experimental results with a theoretical model, it was concluded that ionic liquids behave as weak electrolytes with a dissociation constant of 10^–4^. According to the authors, the vast majority of ions would be paired in ionic liquids. In other words, ILs were somehow like water, which could be viewed as an “ionic liquid” with protons and hydroxides as the cations and anions respectively and the neutral water molecule as the “ion pair” with a dissociation constant of 10^–14^. If this conclusion was correct, it would be very difficult to rationalize many physical properties of ILs, such as their extremely low vapor pressures. Soon after its publication, the work was challenged by multiple groups with theoretical analyses.^7^–^9^ In fact, a series of experimental and theoretical studies including detailed studies on nucleophilic substitutions in ionic liquids,^25^ charge transfer UV spectra of Kosower's salt in ionic liquids,^26^ applications of various techniques to mixtures of ionic liquids,^4^ as well as plentiful computational works^27^,^28^ have suggested ion dissociation in ILs.

Until now, both “ion pairing or clustering” supporters and opponents have found some evidence indirectly favoring each side. However, neither side has direct experimental results *in situ* to reveal the structure of ILs. To resolve this controversy, what is needed is a tool able to directly capture the interactions between cations and anions in the bulk liquid state of ILs with a temporal resolution faster than ion dissociations. In this work, we investigate the local structures of ionic liquids, by directly measuring the relative orientations and vibrational couplings between neighboring counter-ions using a home built high-powered ultrafast multiple-mode two dimensional infrared (2D IR) spectroscopic technique.^29^–^31^ Surprisingly, in contrast to the well-propagated idea of ion pairing, a random orientation between ions was observed in the ionic liquids despite the strong, direct interactions between the non-spherically symmetrical cations and anions.

## Results

Our strategy to investigate whether cations/anions form ion pairs in ILs was to measure the relative orientation between the nearest cations and anions. The relative ionic orientation determines the relative orientations of chemical bonds and vibrational modes between the ions. If two ions completely dissociate, the relative orientation of one ion to its nearest counterion must be random and the average anisotropy is zero. Otherwise, if they form an ion pair, the relative orientation must be directional, provided that neither ion is spherically symmetrical. In experiments, we selectively excited one vibrational mode on one ion, and detected its coupling to vibrational modes on the counterion(s). The excitation and detection pulses were set to be either parallel or perpendicular to each other, and the relative strength of the signals from the two experiments reflects the relative orientation of the two coupled vibrational modes.

**Fig. 1:**
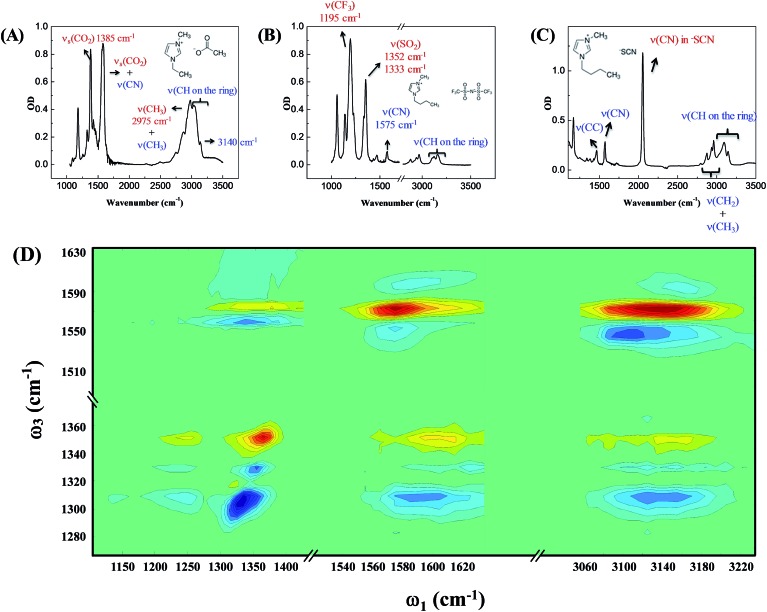
FTIR spectra of (A) 1-ethyl-3-methylimidazolium acetate; (B) 1-butyl-3-methylimidazolium bis(trifluoromethylsulfonyl)imide; (C) 1-butyl-3-methylimidazolium thiocyanate. The peaks were assigned based on comparison with *ab initio* calculations and database results. (D) 2D IR spectrum of 1-butyl-3-methylimidazolium bis(trifluoromethylsulfonyl)imide at time zero.

Three room temperature ILs, 1-ethyl-3-methylimidazolium acetate, 1-butyl-3-methylimidazolium bis(trifluoromethylsulfonyl)imide, and 1-butyl-3-methylimidazolium thiocyanate, were studied in this work. Their molecular structures and FTIR spectra are shown in Fig. 1A–C. In each IL, the cation has vibrational modes (blue) well distinguishable from those of the anion (red). These modes were further interrogated with multiple-mode 2D IR measurements to obtain the relative spatial orientation between the cation and anion. Fig. 1D shows such a 2D IR spectrum of 1-butyl-3-methylimidazolium bis(trifluoromethylsulfonyl)imide. The full spectrum was obtained by exciting vibrational modes from 1100 cm^–1^ to 3250 cm^–1^ (*ω*_1_) and detecting the responses of vibrational modes between 1250 cm^–1^ to 1650 cm^–1^ (*ω*_3_) at a zero delay. The excitation of one vibrational mode shifts the frequency of another mode, resulting in bleaching (red peaks) and absorption (blue peaks) signals, respectively.^32^–^34^

**Fig. 2:**
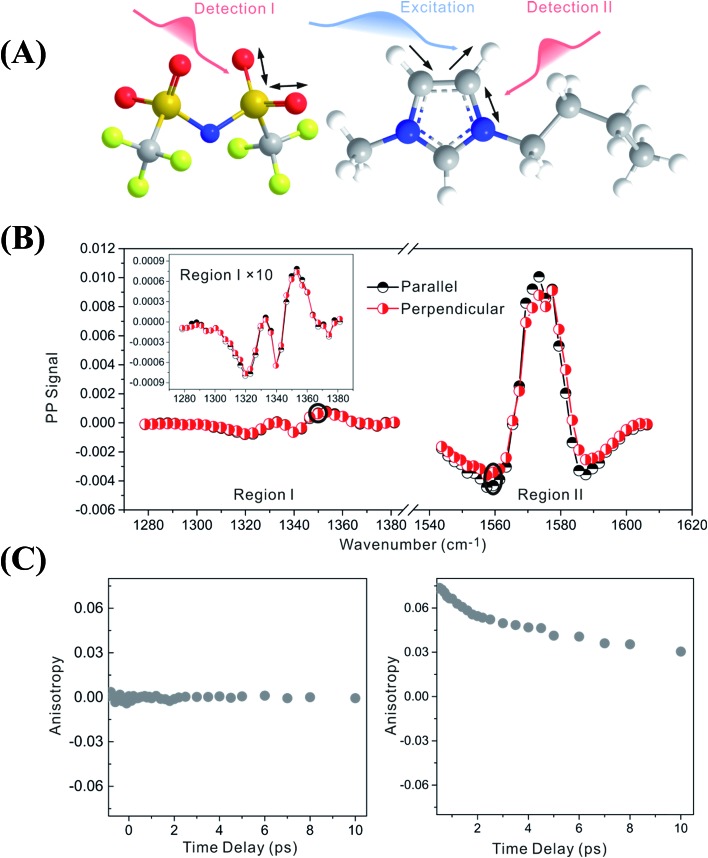
Pump-probe signals and anisotropy data of 1-butyl-3-methylimidazolium bis(trifluoromethylsulfonyl)imide. Upper panel (A): molecular structure. Middle panel (B): vibrational coupling signals measured by exciting the cation at 3160 cm^–1^. When detecting the response of the anion in region I (from 1280 to 1380 cm^–1^), the intensities of the parallel (dark half circles) and perpendicular (red half circles) signals are the same, and therefore the anisotropy value is zero. On the contrary, when detecting the response of the cation in region II (from 1540 to 1610 cm^–1^), the parallel and perpendicular intensities are different. A nonzero anisotropy value, ∼0.07, is detected, corresponding to a cross angle of 48°. All data plotted were measured at a time delay of 0 fs. Lower panels (C): waiting time dependent anisotropy values of the signal 1352 cm^–1^ (left) and 1560 cm^–1^ (right).

Fig. 2 focuses on the data obtained by exciting the cation at 3160 cm^–1^ (ring C–H antisymmetric stretch) and detecting the response of the anion from 1280 to 1380 cm^–1^ (region I in the figure, featuring SO_2_ symmetric and antisymmetric stretches) and that of the cation from 1540 to 1610 cm^–1^ (region II in the figure, featuring the CN stretches on the imidazolium ring). The experiments were carried out using both parallel and perpendicular setups. The parallel spectrum is obtained by setting the polarization of the excitation light to be the same as that of the detection light, and the perpendicular spectrum obtained by setting the two polarizations perpendicular to each other. The comparison of signals from the two polarization setups enables us to obtain the anisotropy,^35^ according to eqn (1):1
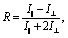
where *I*_‖_ and *I*_⊥_ are the intensities of the parallel and perpendicular signals, respectively. The anisotropy value can be converted into the cross angle *θ* between the excited and detected vibrational modes using eqn (2):2
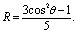



Interestingly, the parallel and perpendicular signals overlap perfectly in the case of interionic coupling (region I of Fig. 2B), yielding zero anisotropy (left panel of Fig. 2C). In stark contrast, the signals in the two polarization setups for the intra–cationic coupling between the CH stretch at 3160 cm^–1^ and the CN stretch at 1560 cm^–1^ are different: the parallel signal is larger in amplitude in both the positive peaks at 1575 cm^–1^ and the negative peaks at 1560 cm^–1^ (region II of Fig. 2B). Quantitative analysis according to eqn (1) and (2) results in the anisotropy value to equal ∼0.07 and the corresponding cross angle between the two modes to be 48 ± 1 degrees. As expected, the anisotropy value gradually decays in a picosecond time scale (right plot in Fig. 2C), due to molecular rotations and vibrational energy transfers, including relaxation-induced thermal energy transfer.^30^,^36^ In comparison, the anisotropy of interionic coupling is zero at the beginning and cannot decay anymore, as shown in the left hand plot of Fig. 2C.

**Fig. 3:**
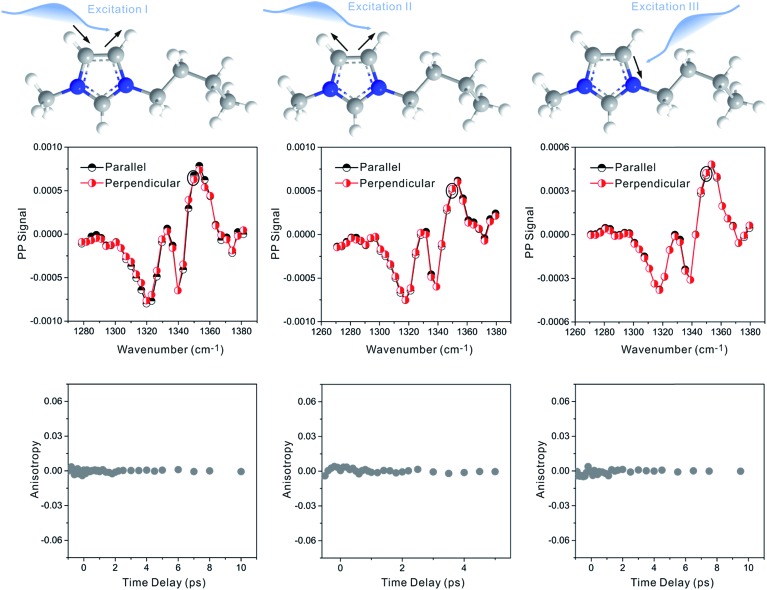
Zero anisotropy of vibrational coupling between the three modes on the cations and the two modes on the anions. Left panel, excited at 3160 cm^–1^, the CH antisymmetric stretch of the cation. Central panel, excited at 3120 cm^–1^, the CH symmetric stretch of the cation. Right panel, excited at 1575 cm^–1^, the CN stretch mode of the cation. All of the signals were detected at the SO_2_ antisymmetric stretch (1352 cm^–1^) and symmetric stretch (1333 cm^–1^) of the anion.

**Fig. 4:**
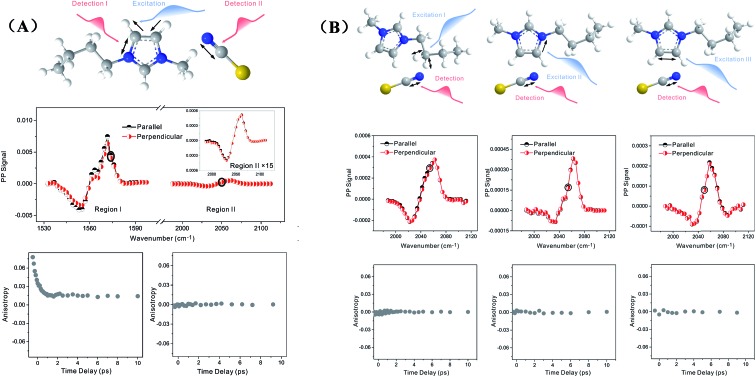
Anisotropy data of 1-butyl-3-methylimidazolium thiocyanate. (A) Upper panel: molecular structure. Middle panel: intraionic (left) and interionic (right) vibrational coupling signals. Lower panels: waiting time dependent anisotropy values of the signals. (B) Upper panel: molecular structure. Middle panel: interionic vibrational coupling signals. Lower panels: waiting time dependent anisotropy values of the signals.

**Fig. 5:**
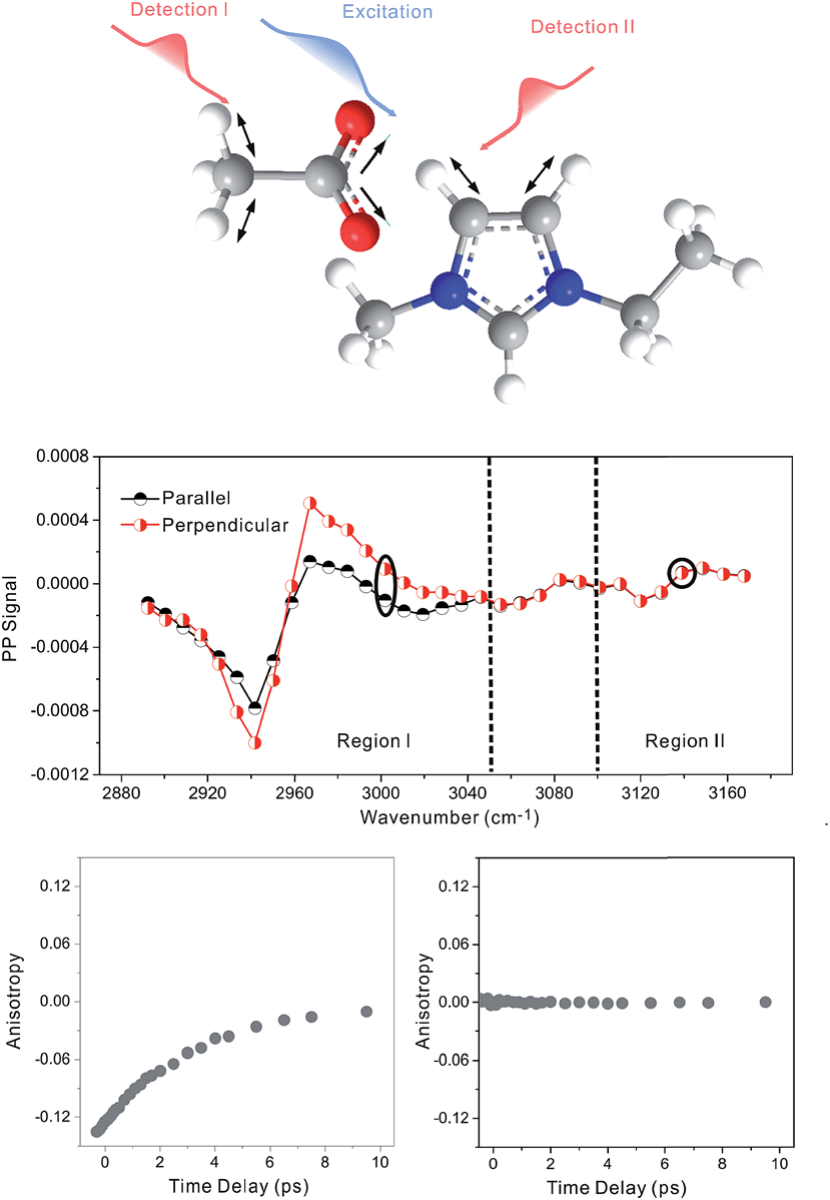
Anisotropy data of 1-ethyl-3-methylimidazolium acetate. Upper panel: molecular structure. Middle panel: intra-ionic (region I on the left) and interionic (region II on the right) vibrational coupling signals. Lower panels: waiting time dependent anisotropy values of the signals.

Zero anisotropy is not limited to between the antisymmetric CH stretch on the cation ring (3160 cm^–1^) and the anion SO_2_ antisymmetric stretch (1352 cm^–1^). As shown in Fig. 3, by exciting any of the three modes on the cations, the cation CH antisymmetric (3160 cm^–1^) and symmetric (3120 cm^–1^) and CN (1575 cm^–1^) stretches, and detecting the anion SO_2_ antisymmetric (1352 cm^–1^) and symmetric (1333 cm^–1^) stretches, always results in signals with the same parallel and perpendicular intensities. Measurements on the vibrational coupling in the other two ILs, 1-ethyl-3-methylimidazolium acetate and 1-butyl-3-methylimidazolium thiocyanate, yield similar results. As displayed in Fig. 4 and 5, in each IL, the anisotropy of interionic coupling is always zero, whereas those of the intra-ionic coupling are not zero, and are time-dependent. As discussed later in detail, such zero anisotropy results demonstrate that interionic orientation is random.

## Discussion

### The lack of anisotropy indicates the isotropic distribution of ions

Experimentally, we observe zero anisotropy in all pairs of interionic vibrational modes investigated. Theoretically, the lack of anisotropy in a pair of two coupling modes could be attributed to two possible origins: first, the cross angle is at the magic angle of 54.7°; and second, the relative orientation of the two modes is random. In our case, the first possibility is extremely low or even impossible. It is very unlikely that the angles happen to be at the magic angle for all pairs of interionic vibrational couplings in three different ILs. Furthermore, the angles in two of the three ILs we studied, 1-butyl-3-methylimidazolium bis(trifluoromethylsulfonyl)imide and 1-butyl-3-methylimidazolium thiocyanate, are geometrically interdependent; it can be rigorously proven that all of them cannot be at the magic angle simultaneously. The details of derivation are provided in the ESI.†

Therefore, the zero anisotropy we measured should be attributed to the random distribution of ions. The experimentally observed anisotropy contain contributions from all possible equilibrium configurations and conformations. Similar to the anisotropy in the fluorescence spectroscopy, the anisotropy in our experiments follows the additivity law, as long as fractional intensities rather than fractional populations are used:3
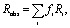
where *R*_*i*_ is the anisotropy of the *i*^th^ component and *f*_*i*_ is its fraction. Given our experimental uncertainty (∼0.01), the zero anisotropy we reported technically means that the true value of the anisotropy must be within ±0.01. In the following, we will elaborate on the meaning of such low anisotropy in ILs.

Firstly, ion pairs, even if they exist in ILs, must be present in very low concentrations. The upper limit of the fraction of ion pairs can be estimated from our experimental uncertainty. In the model of ion pairing, there are two configurations: associated ion pairs and dissociated ones. The overall anisotropy is the weighted average of the two species: *R*_obs_ = *f*_ip_*R*_ip_ + *f*_dis_*R*_dis_. Since the anisotropy of the dissociated species can be assumed to be zero, the equation is further reduced to: *R*_obs_ = *f*_ip_*R*_ip_. For a pair of coupled vibrational modes with a fixed angle of 30°, *R*_ip_ is 0.25; the fraction of the associated ion pair, *f*_ip_, must be as small as 4% to reduce the *R*_obs_ value to 0.01, the experimental uncertainty. When the two coupling modes are perfectly aligned (the angle is 0°), *R*_ip_ is 0.4, and the upper limit of *f*_ip_ further decreases to 2.5%. Of course, with the angle approaching the magic angle, 54.7°, the fraction could in principle become larger. However, as stated earlier, some of the cross angles are geometrically interdependent: if one pair is close to 54.7°, another pair tends to be far away from this angle. The angle interdependency and the experimental uncertainty together ensure that the fraction of ion pairing must be very small in the ILs.

Secondly, ion pairs, even if they exist in ILs, must be very flexible. The experimentally observed anisotropy is the average over multiple conformations. A loosely-bound ion pair would allow relative rotation of an ion in respective to the other. The cross angle between them should adopt a certain distribution, *ρ*(*Ω*) and the overall *R*_obs_ becomes the weighted average of *R*_ip_:4
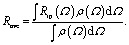



The average value *R*_ave_ deceases as the angular distribution becomes broader. Quantitatively evaluating the effect requires a precise knowledge of *ρ*(*Ω*), which is not easily available. For the purpose of discussion, we will consider two possible distributions, a Gaussian distribution and a Boltzman distribution with a locked dipole model, to estimate the effect of conformational fluctuation on the overall anisotropy. With the details provided in the ESI,† herein we will briefly state the major conclusions. If we assume the angular distribution follows a Gaussian distribution centered at angle *θ* with a width of *σ*, the *R*_ave_ can be calculated numerically. For *θ* = 30°, the *σ* value has to be as large as 59° to reduce *R*_ave_ to 0.01. The degree of fluctuation is about 2/3 of all possible angles (0–90°). If instead, we assume a Boltzman distribution similar to an ion-locked dipole, the interaction energy needs to be as small as 0.9 kcal mol^–1^, which is comparable (Boltzman factor 61%) to the thermal energy 0.6 kcal mol^–1^ at room temperature, to reduce *R*_ave_ to 0.01. Either way, the conformational fluctuation needs to be very large, over 60% of all possible angles, to explain the extremely low anisotropy observed experimentally. By definition, a large interionic conformational fluctuation physically means a very weak ion pairing.

In summary, the lack of anisotropy in any interionic vibrational coupling in ILs cannot be explained by accidentally being at the magic angle. Instead, such a result indicates a nearly random ordering of ions in ILs. Ion pairs and/or ion clusters, if they exist, must be present in very small factions or fluctuate to such a large degree that they would not be regarded as ion pairs or ion clusters, at least not in their classical definition.

### Structure and nanostructure of ILs

**Scheme 1:**
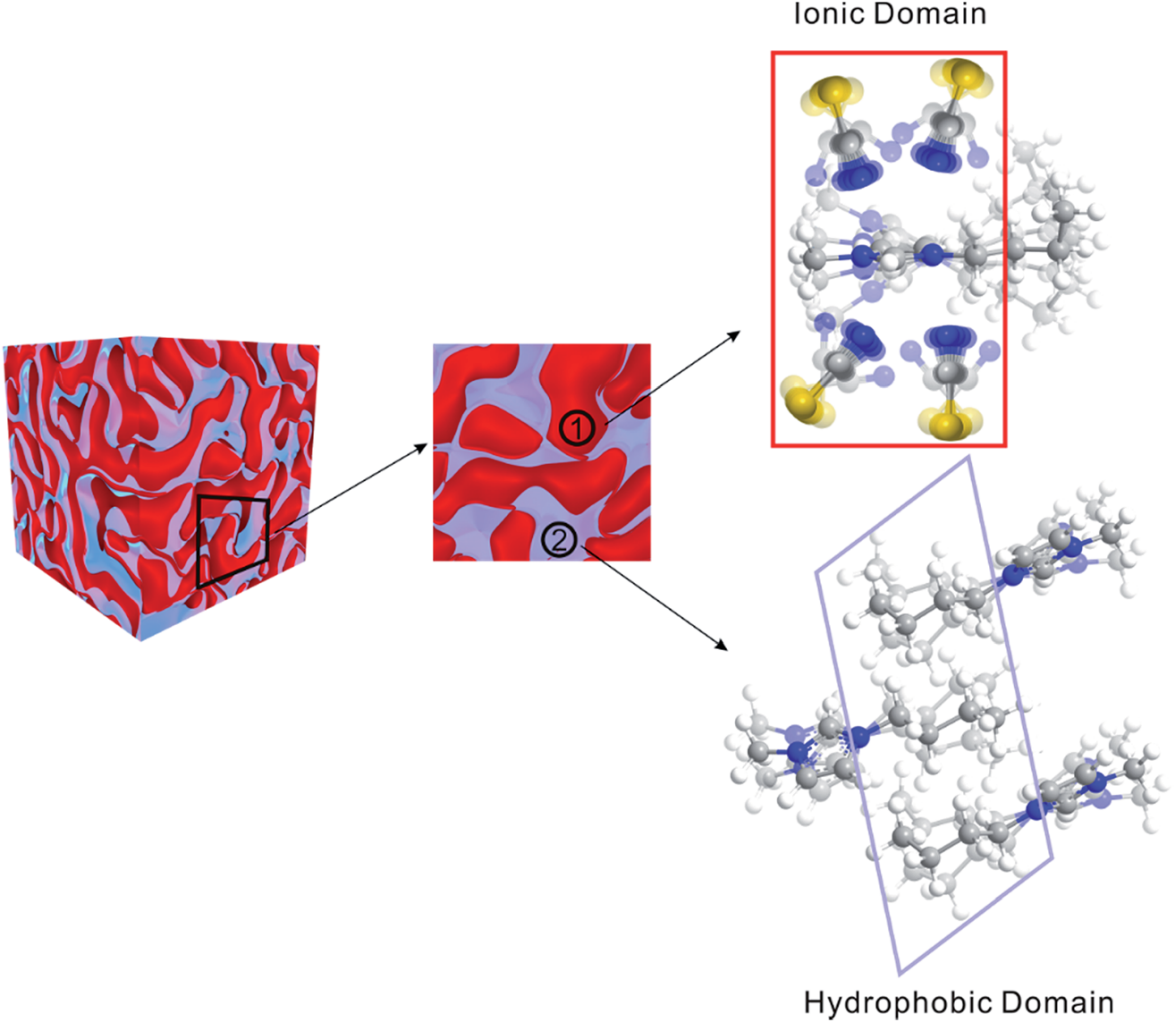
Schematic of structures of ILs. On the mesoscale, ILs form a bicontinuous structure, comprised of ionic domains and hydrophobic domains. In the ionic domain, the interionic orientation is random.

Matter can be structured differently over multiple length scales. The intermolecular vibrational coupling probed in our experiments is only effective over a few angstroms,^17^,^18^ and the conclusion of interionic random orientation is important only for directly-neighboring cations and anions, because the sizes of the molecules/ions studied here are a few angstroms. Over larger length scales, however, accumulating evidence shows most ILs are highly structured over the nanometer length scales. The most compelling evidence comes from X-ray scattering experiments,^37^,^38^ which suggest ionic/alkyl alternative structures with nano-sized primary periodicity. These mesoscopic regions form a bicontinuous, sponge-like nanostructure,^39^,^40^ as shown in Scheme 1. Such structures can be rationalized considering that ILs are both “ionic” and “molecular”. In a typical IL, such as the ones studied here, the ionic functional group (the imidazolium in our case) and hydrophobic functional group (the aliphatic side chains) coexist in one ion, while the counterion is purely ionic. The imidazolium ring interacts strongly with the anion; together they form the ionic part of the ILs. In contrast, the aliphatic side chains interact strongly with neither the imidazolium nor the anion. Due to the great discrepancy in the physical properties between the ionic part and the hydrophobic part, they are spontaneously “phase-separated” into mesoscopic domains. The X-ray scattering experiments and vibrational coupling experiments are complementary to each other. The X-ray experiments suggest an ordered ionic/hydrophobic phase separation at the mesoscale, whereas our experiments prove the ordering of ions inside the ionic domain at the molecular scale is virtually random, similar to molten salts.

In such a heterogeneous structure of ILs, the average distance between cations and anions is closer than one might expect solely from their concentration. The proximity guarantees strong cation/anion interactions, which could provide a possibility for a high degree of charge transfer between counter ions.^22^,^23^ Charge transfer between cations/anions would result in a partial charge for both ions and significantly lower the overall ionic conductivities.^22^,^23^ The “unexpectedly low” experimental values of the bulk ionic conductivities of ILs might be simply because their values were overestimated when assuming unit charges.^22^,^23^

In summary, ILs are highly ordered on the mesoscale (nanometer), but virtually randomly distributed on the molecular scale (sub-nanometer) in the ionic domains and hydrophobic domains. Such a structure is rare in liquids. One might regard ILs as the “mixture” of two continuous domains: a molten-salt-like ionic domain, which is essentially a highly flexible ion cluster, but without well-defined structures like those seen in the gas phase;^13^,^38^,^41^ and a molecular domain, which is similar to nonpolar molecular liquids. Therefore, ILs are not the “intermediate” between molten salts and molecular liquids. In this sense, they are more like a 3D interconnecting “nanocomposite” of molten-salt-like domains and molecular-liquid-like domains with covalent bonds connecting them, as illustrated in Scheme 1. The strong electrostatic interactions between the dissociated ions in the ionic domains contributes to extremely low vapor pressure, and the weak van de Waals forces in the molecular domains contribute to low melting point. As in many nanocomposite materials, ILs often possess the composite favorable properties of both components, rather than just the “mediocre” properties of simple mixtures of component materials. We also expect that the high-powered 2D IR technique demonstrated here, that can detect extremely weak intermolecular interactions, will find much wider applications in various fields far beyond physical chemistry.

## Method

The 2D IR spectroscopy setup has been described in detail previously.^29^–^31^ Briefly, the excitation pulse was a mid-IR beam with a pulse duration of ∼0.8 ps, a bandwidth of 10–35 cm^–1^, and repetition rate of 1 kHz. It was generated using a ps, 800 nm amplifier pumping an OPA (optical parametric amplifier). Its frequency was tunable, ranging from 400 to 4000 cm^–1^, and the pulse energy reached 1–40 μJ pulse^–1^ (>10 μJ pulse^–1^ in the frequency range of 1000 to 4000 cm^–1^ for experiments herein). The detection beam is a high-intensity mid-IR and terahertz supercontinuum beam, generated by an fs amplifier that is synchronized with the ps amplifier, using the same seed pulse from the same oscillator. Specifically, the collimated 800 nm beam from the fs amplifier is frequency-doubled by passing through a type-I 150 μm thick BBO crystal to generate a 400 nm beam. The polarization difference between the 800 and 400 nm pulses was corrected using a dual wave plate which operated as a full-wave plate at 400 nm and a half-wave plate at 800 nm. The temporal walk off between the two beams was compensated by inserting a delay plate between the doubling crystal and the wave plate. The supercontinuum was then obtained by focusing the two copropagating beams on air, resulting in a pulse with duration around 110 fs in the frequency range from <20 to >3500 cm^–1^, and the shot to shot fluctuation was less than 1% in most of the spectral region. In the 2D IR experiments, the excitation beam was focused on the sample with an interaction spot varying from 100 to 500 μm; the power was adjusted based on need. The detection beam, after passing the same spot, was frequency-resolved using a spectrograph (the resolution was 1–3 cm^–1^ dependent on the frequency) to yield the detection axis of a 2D IR spectrum. Scanning the excitation frequency over the range of interest yields the other axis of the spectrum. Two polarizers in the detection beam path enabled us to selectively measure the parallel or perpendicular polarized signal relative to the excitation beam. The whole setup, including frequency tuning, was computer controlled using a home-written program. The three ILs, 1-ethyl-3-methylimidazolium acetate, 1-butyl-3-methylimidazolium bis(trifluoromethylsulfonyl)imide, and 1-butyl-3-methylimidazolium thiocyanate, were purchased from Aldrich, and used as received. The ILs were loaded in the CaF_2_ window as the sample holder to obtain the 2D IR spectra.

The molecular structures and relative cross angles between the different vibrational modes were determined with density functional theory (DFT) calculations. The DFT calculations were carried out using Gaussian 09. The level and basis set used were Becke's 3-parameter hybrid functional combined with the Lee–Yang–Parr correction functional (B3LYP) and 6-311++G(d,p), respectively.

## Competing financial interests

The authors declare no competing financial interests.

## Conflicts of interest

There are no conflicts to declare.

## Supplementary Material

Supplementary information
