# Supplementary material for: Isotropic ordering of ions in ionic liquids on the sub-nanometer scale
Source: Chem Sci. 2017 Dec 22;9(6):1464–72. doi: 10.1039/c7sc05184k (PMC5890800; doi:10.1039/c7sc05184k)
Supplement: Supplementary file 1 [file SC-009-C7SC05184K-s001.pdf]

Electronic Supplementary Material (ESI) for Chemical Science.
This journal is © The Royal Society of Chemistry 2017

Isotropic Ordering of Ions in Ionic Liquids on the Sub-Nanometer Scale

Hailong Chen[2†], Xin Chen[1†], Jingwen Deng[1], Junrong Zheng[1*]

1 College of Chemistry and Molecular Engineering, Beijing National Laboratory for Molecular Sciences, Peking University, Beijing 100871, China

2 Beijing National Laboratory for Condensed Matter Physics, CAS Key Laboratory of Soft Matter Physics, Institute of Physics, Chinese Academy of Sciences, Beijing 100190, China

† These authors contributed equally to the work

* To whom correspondence should be addressed: junrong@pku.edu.cn, and zhengjunrong@gmail.com

1. Geometric Interdependence of the angles in vibrational coupling

1.1 1-Butyl-3-methylimidazolium bis(trifluoromethylsulfonyl)imide

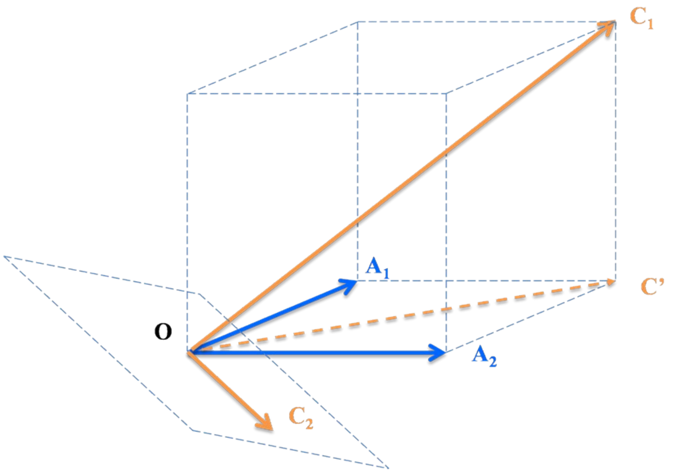

Figure S1. Illustration of two pairs of perpendicular vectors. $\overrightarrow{OA_1}$ and $\overrightarrow{OA_2}$ are perpendicular to each other, and $\overrightarrow{OC_1}$ and $\overrightarrow{OC_2}$ are perpendicular to each other. As explained in the text, the four angles formed by such four vectors cannot be at the magical angle simultaneously.

As illustrated in Fig.S1, the angles in vibrational coupling are often interdependent. In 1-Butyl-3-methylimidazolium bis(trifluoromethylsulfonyl)imide, there are two vibrational modes of SO_2 functional group on the anion: the antisymmetric stretch at 1352 cm^{-1} and symmetric stretch at 1333 cm^{-1} (shown as the vectors $\overrightarrow{OA_1}$ and $\overrightarrow{OA_2}$ in the figure). By definition, their dipole moments are perpendicular to each other ($\angle A_1OA_2 = 90^o$). Similarly, there are two vibrational modes of CH on the cationic ring: the antisymmetric stretch at 3160 cm^{-1} and the CH symmetric stretch at 3120 cm^{-1} (shown as the vectors $\overrightarrow{OC_1}$ and $\overrightarrow{OC_2}$ in the figure), which are also perpendicular to each other ($\angle C_1OC_2 = 90^o$).

The four angles formed by such four vectors are interdependent and cannot be at a certain angle simultaneously. For example, if $\angle C_1OA_1 = \angle C_1OA_2 = 54.7°$, the angle between the $\overrightarrow{OC_1}$ and the A_1OA_2 plane, the $\angle C_1OC'$, must be 35°:

$$\angle C_1OC' = \arccos\left(\frac{\cos(45°)}{\frac{1}{\cos(54.7°)}}\right) = 35°$$

Similarly, if $\angle C_2OA_1 = \angle C_2OA_2 = 54.7°$, the $\angle C_2OC'$, must be 35° too. This would contradict with the limit condition $\angle C_1OC_2 = 90°$.

1.2 1-butyl-3-methylimidazolium thiocyanate

In 1-butyl-3-methylimidazolium thiocyanate, three vibrational modes 1465 cm^{-1}, 1572 cm^{-1} and 3095 cm^{-1} are mainly located on the anion ring according to DFT calculations. As shown in fig.S2, transition dipole moments of these three cation modes are almost on the same plane (53°+80°+49°≈180°). Our experimental results demonstrate that the relative orientation between any one of these three cation modes and the CN stretch mode of anion (2053 cm^{-1}) is either random or equal to 54.7°. Obviously, it is impossible that all cross angles between three cation modes and one anion mode are 54.7°, or only two of them are 54.7° with the third one is a random angle. For example, if we fix both cross angles (1572 cm^{-1}, 2053 cm^{-1}) and (1465 cm^{-1}, 2053 cm^{-1}) to be 54.7°. The cross angle (3095 cm^{-1}, 2053 cm^{-1}) should be close to 88°, rather than 54.7° or a random value. Moreover, if only one of these three angles is equal to 54.7°, then the other cross angles cannot be random either. For example, if we fix cross angles (1572 cm^{-1}, 2053 cm^{-1}) to be 54.7°. The cross angle (1465 cm^{-1}, 2053 cm^{-1}) should be larger than 25°, and therefore cannot be a random value. Similarly, the cross angle (3095 cm^{-1}, 2053 cm^{-1}) cannot be

random either. As a result, the only possibility is that the relative orientation between any cation mode and anion mode is random.

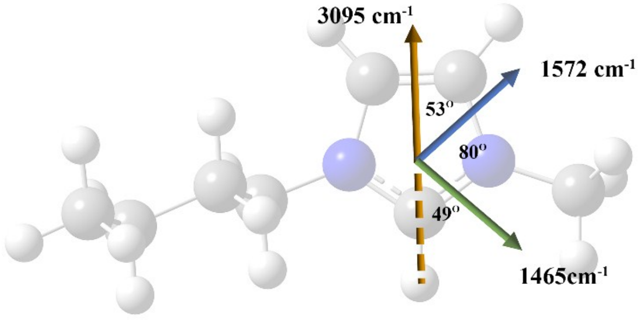

Figure S2. Illustration of calculated orientations of three cation modes as well as calculated cross angles among them.

2. Estimations of the flexibility of ion pairing

In the classic definition, an ion pair adopts a well-defined configuration, as shown in fig.S3a. However, in reality, the ion pair could be more flexible, such as shown in fig.S3b. Herein we could estimate the degree of flexibility based on the anisotropy of the interionic vibrational coupling of the two ions.

The anisotropy in our experiments follows the additivity law: $R = \sum_i f_i R_i$, where R_i is the anisotropy of *ith* component and f_i is the its fraction in intensity, not its fraction in population. In case of continuous distribution, the overall R_{obs} become the weighted average of R_{ip}:

$$R = \frac{\int R_{ip}(\Omega) * f(\Omega) d\Omega}{\int f(\Omega) d\Omega}, \qquad \text{(eq.S1)}$$

where $\rho(\Omega)$ is the distribution function of the cross angle.

If we assume the angular distribution can be described with a Gaussian function centered at angle θ with a width of σ, the R_{ave} can be calculated numerically. Set one vibrational mode on one ion fixed in space, then the most stable conformation of the other mode on the other ion must sit at an angle θ relative to the first mode. For a particular conformation of the second mode with tilting angle φ and twist angle ϕ relative to the most stable conformation, its titling angle relative to the first mode is $\theta' = \theta + \varphi * \cos(\phi)$. Thus, the average anisotropy can be calculated by evaluating the equation:

$$R_{ave}(\theta,\sigma) = \frac{\int R_{ip}(\theta') * G(\varphi) d\Omega}{\int G(\varphi) d\Omega} = \frac{\int_{\phi=0}^{2\pi}\int_{\varphi=0}^{\pi} R_{ip}(\theta + \varphi\cos(\phi)) * \exp\left(-\frac{\varphi^2}{2\sigma^2}\right) d\varphi d\phi}{\int_{\phi=0}^{2\pi}\int_{\varphi=0}^{\pi} \exp\left(-\frac{\varphi^2}{2\sigma^2}\right) d\varphi d\phi}.$$

(eq.S2)

For example, if θ = 30°, the σ value has to be as large as 59° to reduce R_{ave} to 0.01. This means that the degree of fluctuation of the angle is even greater than the angular orientation, and this amplitude is about 2/3 of all possible angles 0-90^0.

If instead we assume the angular distribution is a Boltzman distribution in a locked dipole model with the interaction free energy ΔG, at room temperature T, the R_{ave} can be calculated numerically using the equation:

$$R_{ave}(\theta,\Delta G) = \frac{\int_{\phi=0}^{2\pi}\int_{\varphi=0}^{\pi} R_{ip}(\theta + \varphi cos(\phi)) * exp(\frac{-\Delta G * \cos(\theta + \varphi cos(\phi))}{RT})\, d\varphi d\phi}{\int_{\phi=0}^{2\pi}\int_{\varphi=0}^{\pi} exp(\frac{-\Delta G * \cos(\theta + \varphi cos(\phi))}{RT})\, d\varphi d\phi}.$$

(eq.S3)

For example, if θ = 30°, the ΔG value has to be as small as ~0.9 kcal/mol to reduce R_{ave} to 0.01. This value is comparable with the thermal energy at room temperature with a Boltzman factor 0.61; the interactions must be very weak and the degree of fluctuation very large. Either way, the ion pairs, if exist, must be very flexible to yield an anisotropy value as low as 0.01. In other words, the orientation of ions must be very close to isotropic.

Similar arguments could be applied to ion clustering as well, since an ion cluster could be simply regarded as a collation of a few ion pairs. In theory, the anisotropy of an ion cluster could be treated as the weighted average of several ion pairs. However, since we do not know the exact configuration of the ion cluster, we cannot evaluate the anisotropy numerically. Nevertheless, we can conclude that a highly flexible ion cluster, such as the one shown in fig.S3d, would show very small anisotropy. In fact, such a structure is not fundamentally different from an ionic domain with completely random distributed ions, as shown in fig.S3e.

Lastly, we note that these calculations should be treated as estimations rather than rigorous calculations, for a couple of reasons: first, we do not know the distribution precisely, and second, we used the fraction in populations instead of the fraction in intensity, which is obviously an approximation. Nevertheless, we are trying to estimate the degree of fluctuation of the ion pairs/ion clusters, and these practices clearly show that the

ion pairs/ion clusters must be in very low concentrations (<10~20% at possibly most) and/or highly flexible to generate anisotropy smaller than 0.01, so that we cannot measure their values experimentally.

(A) A classic ion pair	**(B)** A soft ion pair
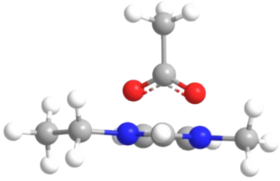	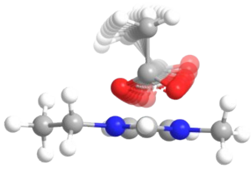
(C) A classic ion cluster	**(D)** A soft ion cluster
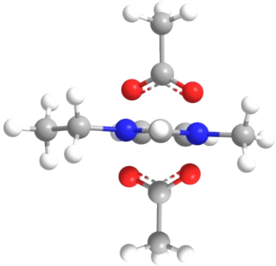	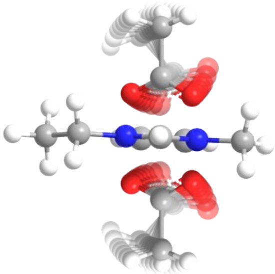
(E) An ionic domain with random ion distribution	
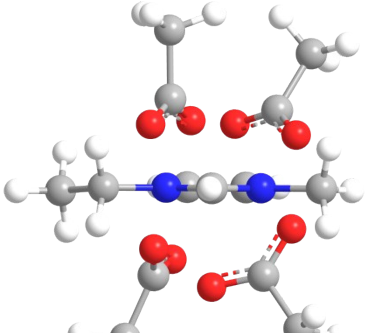	

Figure S3. Schematic of a classic ion pair, a soft ion pair, a classic ion cluster, a soft ion cluster, and an ionic domain with random distribution of ions.
